# microRNA-21 Regulates Stemness in Pancreatic Ductal Adenocarcinoma Cells

**DOI:** 10.3390/ijms23031275

**Published:** 2022-01-24

**Authors:** Maria Mortoglou, Francesc Miralles, Elif Damla Arisan, Alwyn Dart, Stipo Jurcevic, Sigrun Lange, Pinar Uysal-Onganer

**Affiliations:** 1 Cancer Research Group, School of Life Sciences, University of Westminster, London W1W 6UW, UK; w1754188@my.westminster.ac.uk; 2 Molecular and Clinical Sciences, Cell Biology Research Centre, St. George’s University of London, Cranmer Terrace, SW17ORE London, UK; fmiralle@sgul.ac.uk; 3 Institute of Medical and Biomedical Education, St. George’s University of London, Cranmer Terrace, London SW17 ORE, UK; ddart@sgul.ac.uk; 4 Institution of Biotechnology, Gebze Technical University, Gebze 41400, Turkey; damlaarisan@gmail.com; 5 Department of Biomedical Sciences, School of Life Sciences, University of Westminster, London W1W 6UW, UK; s.jurcevic@westminster.ac.uk; 6 Tissue Architecture and Regeneration Research Group, School of Life Sciences, University of Westminster, London W1W 6UW, UK; s.lange@westminster.ac.uk

**Keywords:** pancreatic ductal adenocarcinoma, microRNAs, non-coding RNAs, cancer stem cells, metastasis, epithelial–mesenchymal transition

## Abstract

Pancreatic ductal adenocarcinoma (PDAC) is the most common and aggressive type of pancreatic cancer (PCa) with a low survival rate. microRNAs (miRs) are endogenous, non-coding RNAs that moderate numerous biological processes. miRs have been associated with the chemoresistance and metastasis of PDAC and the presence of a subpopulation of highly plastic “stem”-like cells within the tumor, known as cancer stem cells (CSCs). In this study, we investigated the role of miR-21, which is highly expressed in Panc-1 and MiaPaCa-2 PDAC cells in association with CSCs. Following miR-21 knockouts (KO) from both MiaPaCa-2 and Panc-1 cell lines, reversed expressions of epithelial–mesenchymal transition (EMT) and CSCs markers were observed. The expression patterns of key CSC markers, including CD44, CD133, CX-C chemokine receptor type 4 (CXCR4), and aldehyde dehydrogenase-1 (ALDH1), were changed depending on miR-21 status. miR-21 (KO) suppressed cellular invasion of Panc-1 and MiaPaCa-2 cells, as well as the cellular proliferation of MiaPaCa-2 cells. Our data suggest that miR-21 is involved in the stemness of PDAC cells, may play roles in mesenchymal transition, and that miR-21 poses as a novel, functional biomarker for PDAC aggressiveness.

## 1. Introduction

Pancreatic ductal adenocarcinoma (PDAC) is the eighth primary source of cancer-related deaths globally with a 5-year survival rate of 3–6% [1,2,3,4]. Approximately, 10–20% of PDAC patients are compatible for surgery at the time of diagnosis, and 9.7% of PDAC cases are at a local stage when initially diagnosed [5]. microRNAs (miRs) are 18 to 24 nucleotides-long, endogenous, non-coding, evolutionarily conserved, single-stranded RNA molecules. miRs can moderate gene expression at the posttranscriptional level through the binding to the complementary sequences of their target mRNAs at the 3′ untranslated regions (UTRs), which allow them to control the expression levels of several genes and regulate various signaling pathways [6,7,8,9]. Preliminary studies have suggested a correlation between aberrant expression levels of numerous miRs with PDAC [10,11,12,13]. miRs can act as oncogenic miRs (oncomiRs) or tumor suppressor miRs. Especially in PDAC, miR-21, miR-155, and miR-221 have been found to act as oncomiRs, while miR-126 and miR-375 were shown to act as tumor suppressors miRs [14,15]. miR-21, miR-221, and miR-155 can distinguish cases of PDAC from healthy individuals with a sensitivity of approximately 64% and a specificity of 89% [16,17]. Importantly, miRs present a higher sensitivity as a diagnostic marker than the current diagnostic marker carbohydrate antigen (CA 19-9), especially for the early diagnosis of PDAC [18,19,20,21].

Cancer stem cells (CSCs) are involved in chemoresistance and play critical roles in the metastasis of several cancers, including PDAC [22,23,24,25,26,27,28,29]. CSCs contribute to elevated expression levels of anti-apoptotic proteins, ABC transporters, and multidrug resistance genes, high autophagic flux that leads to microenvironment stresses [30,31,32,33,34]. Pancreatic CSCs (PCSCs) are less than 1% of all pancreatic cancer cells and are critical mediators of PDAC tumor growth, maintenance, metastasis, and chemoresistance [35]. EMT is characterized as a critical mechanism of the metastatic cascade, which includes the loss of cell adhesion, elevated cell motility, the repression of E-cadherin, and the upregulation of mesenchymal markers, such as Vimentin, N-cadherin, Snail, and Zeb1 [36]. E-cadherin downregulation is associated with poor prognosis, differentiation, and chemoresistance in PDAC [37,38,39,40]. Transcription marker Zeb1 suppresses E-cadherin through the repression of both miR-203 (an inhibitor of stemness) and miR-200 family members, which control the expression levels of stem cell factors [41]. Zeb1 overexpression is linked to advanced PDAC stages and poor malignancy outcome, migration, and invasion in response to nuclear factor kappa-light-chain-enhancer of activated B cells (NF-κB) signaling [42,43,44]. Non-canonical Wnt-11 overexpression is associated with poor prognosis and tumor-node-metastasis (TNM) staging in PDAC [45,46,47,48,49]. 

Higher expression levels of Snail, a potent EMT-inducing transcription factor, are related to 80% of PDAC cases, E-cadherin downregulation, lymph node invasion, higher tumor grade, and poorly differentiated PDAC cells [50]. EMT is regulated via molecular pathways linked to oncogenic and tumor suppressor non-coding RNAs, chromatin remodeling, epigenetic and posttranslational modifications, alternative splicing events, and protein stability [51,52]. Vimentin is an essential marker of EMT and associated with Notch and miR-200 expression levels and it can affect treatment response in vitro, including elevated gemcitabine-resistance in PDAC [53,54]. Overexpression of Vimentin is linked to metastasis and poor overall survival in PDAC [55,56]. Furthermore, the association between EMT and CSCs has been extensively evaluated, for instance, PDAC cells, which have undergone EMT, and express epithelial markers, such as E-cadherin and mesenchymal markers, such as Zinc Finger E-Box Binding Homeobox 1 (Zeb1), and Snail exhibit stem cell properties [57,58]. The main markers of PCSCs are CD133, CD24, CD44, ESA/EpCAM (epithelial-specific antigen), c-Met, ALDH1, DclK1, CXCR4, and Lgr5 [30,59,60]. Importantly, EMT and autophagy processes are also closely linked with CSCs markers during PDAC development [59,60]. Recent reports stated that CD24, CD44, CXCR4, ESA, and nestin are upregulated in advanced pancreatic intraepithelial neoplasia (PanIN) grades [61], while others have shown that the expression of cMet^+^ CD133^+^ CD34^+^ CD45^−^ Ter119^−^, Pdx1, CD9, CD24, CD44, CD13, and CD133, are linked to poor prognosis of PDAC [61,62,63,64,65,66,67].

miR-21 is one of the most oncogenic miRs related to PDAC prognosis; overexpression of miR-21 was detected in PDAC patients and correlated with poor prognosis and overall survival according to the TCGA dataset (Figure S1). Therefore, the role of miR-21 in PDAC stemness was examined in this study in-depth, using CRISPR-mediated KO approaches in vitro.

## 2. Results

In summary, using three different PDAC cell lines, we found that miR-21, miR-221, miR-155, and miR-126 expressions were significantly altered in MiaPaca-2, Panc-1, and BxPC3 PDAC cell lines, compared with normal pancreatic ductal epithelial cell lines (HPDE). Following the knockout of miR-21 in Panc-1 and MiaPaca-2 cells using CRISPR-Cas9, reversed expressions of E-cadherin, Vimentin, Snail, Wnt-11, and Zeb1 were detected, suggesting that these markers are targets of miR-21. Expression levels of the CSC markers, such as CD133, CD44, CD24, CXCR4, and ALDH1, were significantly downregulated depending on miR-21 status. KO of miR-21 led to a significant reduction in cellular invasiveness of Panc-1 and MiaPaCa-2 cells and a significant decrease in cellular proliferation of MiaPaCa-2 cells. Overall, our data suggest that miR-21 is involved in the pathophysiology of PDAC.

### 2.1. Expression Profiles of miR-21, miR-221, miR-155, and miR-126 in PDAC In Vitro

**Figure 1:**
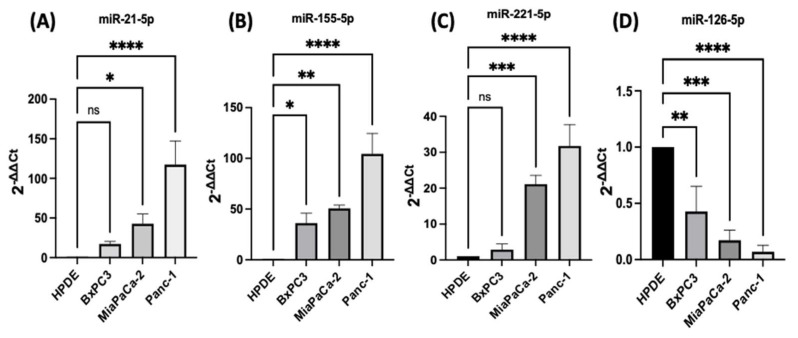
RT-qPCR analysis of miRs levels miR-21, miR-221, miR-155, and miR-126 in PDAC cell lines (BxPC3, MiaPaCa-2, Panc-1, compared with HPDE). (**A**) miR-21-5p relative expression was significantly increased in MiaPaCa-2 and Panc-1 but not in BxPC3 PDAC cell lines. (**B**) miR-155-5p relative expression was significantly overexpressed in BxPC3, MiaPaCa-2, and Panc-1 PDAC cell lines. (**C**) miR-221-5p relative expression was significantly upregulated in MiaPaCa-2 and Panc-1 but not in BxPC3. The column graphic represents the average of three replicates of RNA isolated from each cell line. (**D**) miR-126-5p relative expression was significantly reduced in PDAC cell lines. The column graphs represent the average of three replicates of RNA isolated from each cell line. Data normalized according to RNU6 expression by fold analysis (*n* = 3, *p* < 0.05 for all). *p*-values are indicated as: * *p* ≤ 0.05; ** *p* ≤ 0.01; *** *p* ≤ 0.001; **** *p* ≤ 0.0001; error bars indicate standard deviation (SD).

PDAC associated miR-21, miR-155, miR-221, miR-155, and miR-126 expression levels were quantified using RT-qPCR in three different PDAC cell lines (MiaPaCa-2, Panc-1, and BxPC3), compared with human pancreatic ductal epithelial (HPDE), which served as the control cell line. The HPDE cells had the most epithelial properties expressed relatively, with the least miR-21-5p, miR-155-5p, and miR-221-5p and this was used as a ‘baseline’ (Figure 1A–C). Out of the cell lines tested, Panc-1 cells expressed the highest levels of miR-21-5p (117-fold elevation; *n* = 3; *p* < 0.0001; Figure 1A), miR-155-5p (105-fold increase; *n* = 3; *p* < 0.0001; Figure 1B), and miR-221-5p (32-fold upregulation; *n* = 3; *p* < 0.0001; Figure 1C) relatively compared with HPDE cells. Similarly, MiaPaCa-2 cells presented a 43-fold increase in the miR-21-5p (*n* = 3; *p* < 0.05; Figure 1A), a 51-fold overexpression in miR-155-5p (*n* = 3; *p* < 0.01; Figure 1B) and a 21-fold significant rise in miR-221-5p (*n* = 3; *p* < 0.001; Figure 1C). BxPC3 cells did not show significant changes in both miR-21-5p and miR-221-5p expressions (16-fold; 3-fold, respectively; *n* = 3; *p* > 0.05 for both; Figure 1A,C), however, they showed a 36-fold significant upregulation in miR-155-5p expression (*n* = 3; *p* < 0.05; Figure 1B). The expression levels of miR-126-5p indicated a 57-fold downregulation in BxPC3 cells (*n* = 3; *p* < 0.01; Figure 1D), a 81-fold decrease in the MiaPaCa-2 cell line (*n* = 3; *p* < 0.001; Figure 1D), and a 94-fold reduction in Panc-1 cells (*n* = 3; *p* < 0.0001; Figure 1D) in comparison with normal HPDE cells.

### 2.2. Development of miR-21 KO MiaPaca-2 and Panc-1 Cell Lines

**Figure 2:**
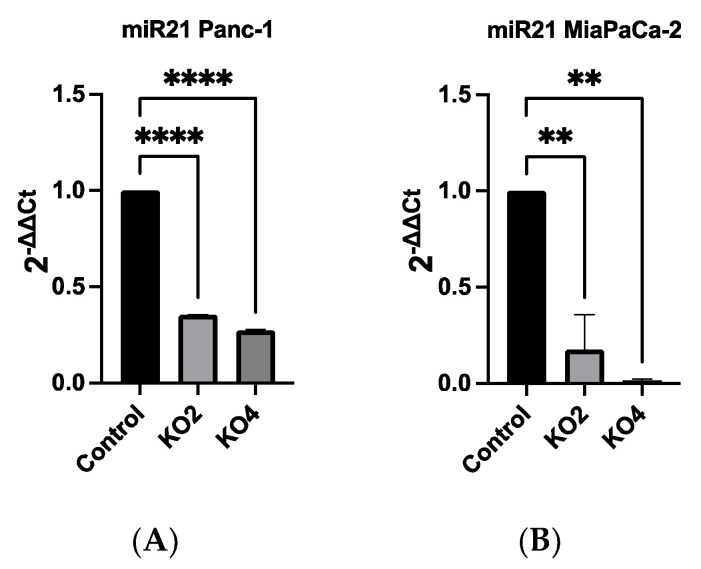
miR-21 expression levels are significantly reduced in Panc-1 and MiaPaCa-2 Kos. (**A**) Relative expression levels of miR-21 in miR-21 KO Panc-1 cells. miR-21 expression levels of miR-21 knockout clone 2 (KO2) and miR-21 knockout clone 4 (KO4) cell colonies were downregulated significantly compared to untreated control Panc-1 cells (*n* = 3; *p* < 0.0001 for all). The column graphic represents the average of three replicates of RNA isolated from the Panc-1 control and its Kos. Data normalized according to RNU6 expression by fold analysis (*n* = 3, *p* < 0.05). (**B**) Relative expression levels of miR-21 in miR-21 KO MiaPaCa-2 cells. miR-21 expression levels of miR-21 knockout clone 2 (KO2) and miR-21 knockout clone 4 (KO4) cell colonies were reduced significantly compared to untreated control MiaPaCa-2 cells (*n* = 3; *p* < 0.01 for all). The column graphic represents the average of three replicates of RNA isolated from MiaPaCa-2 control and its Kos. Data normalized according to RNU6 expression by fold analysis (*n* = 3, *p* < 0.05). *p*-values are indicated as: ** *p* ≤ 0.01; **** *p* ≤ 0.0001; error bars indicate SD.

Our qRT-PCR data showed that miR-21 expression was the most elevated in the Panc-1 and MiaPaCa-2 PDAC cell lines correlating with in vivo data (Figure S1). Therefore, both Panc-1 and MiaPaca-2 were used to further investigate the effect of knocking out miR-21, and cells were transduced with four different miR-21 gRNAs, as well as a control vector. miR-21 expression analysis showed that miR-21 expression was significantly reduced by 65 and 73-fold in Panc-1 knockout (KO) clones 2 (KO2) and 4 (KO4), respectively, compared with Panc-1 vector alone (*n* = 3; *p* < 0.0001 for all; Figure 2A). Similarly, expression levels of miR-21 were also significantly decreased by 83-fold in KO2 and 97-fold in KO4 (*n* = 3; *p* < 0.01 for all; Figure 2B) in MiaPaCa-2. The knockout miR-21 Panc-1 and MiaPaCa-2 PDAC cell lines were further assessed for EMT, and CSC markers and cellular invasion.

### 2.3. miR-21 Kos Reduce Expression Levels of EMT-Related Markers

**Figure 3:**
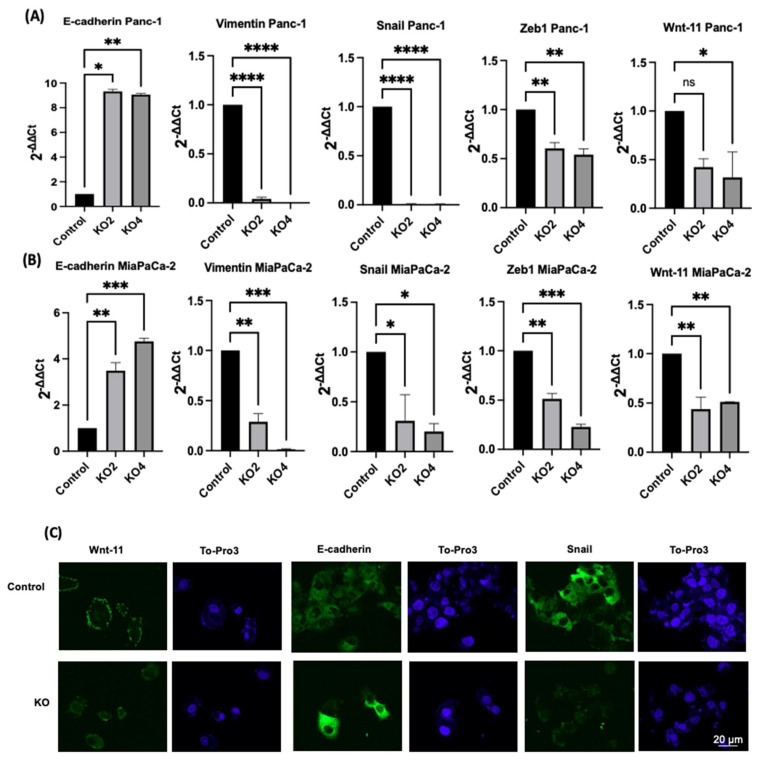
The silencing of miR-21 reduced the mesenchymal phenotype of PDAC cells. (**A**) E-cadherin, Vimentin, Snail, Wnt-11, and Zeb1 in the Panc-1 cell line relative mRNA expression levels. Relative mRNA expression levels of Vimentin, Snail, and Zeb1 were significantly downregulated in miR-21 KO2 and KO4 in the Panc-1 cell line, while E-cadherin was significantly upregulated. Relative mRNA expression levels Wnt-11 were significantly reduced in miR-21 KO4 but not in miR-21 KO2 in the Panc-1 cell line. The column graphic represents the average of three replicates of RNA isolated from Panc-1 control and its Kos. Data normalized according to RNA polymerase II (RPII) expression by fold analysis (*n* = 3, *p* < 0.05). (**B**) Relative mRNA expression levels of E-cadherin, vimentin, Snail, Wnt-11, and Zeb1 in the MiaPaCa-2 cell line. Relative mRNA expression levels of vimentin, Snail, Zeb1, and Wnt-11 significantly decreased in miR-21 KO2 and KO4 in the MiaPaCa-2 cell line, while E-cadherin significantly increased. The column graphic represents the average of three replicates of RNA isolated from MiaPaCa-2 control and its Kos. Data normalized according to RNA polymerase II (RPII) expression by fold analysis (*n* = 3, *p* < 0.05). Exact *p*-values are indicated (* *p* ≤ 0.05; ** *p* ≤ 0.01; *** *p* ≤ 0.001; **** *p* ≤ 0.0001); error bars indicate SD. (**C**) Immunofluorescence assays were performed to show Wnt-11, E-cadherin, Snail protein levels, and protein localization (green) in Panc-1 wt (Control) and miR-21 KO cells. To-Pro3 (blue) was used for staining nuclei. KO2 immunofluorescence results are shown as a representative to compare to control (*n* = 3), the scale bar represents 20 μm in all images.

The effects of miR-21 KO on regulation of key EMT was assessed using miR-21 KO in Panc-1 and MiaPaCa-2 PDAC cells. E-cadherin mRNA expression levels were found to be significantly overexpressed by 932-fold in Panc-1 miR-21 KO2 and by 906-fold in KO4 (*n* = 3; *p* < 0.05 and 0.01, respectively; Figure 3A). Vimentin mRNA levels decreased by 96-fold in KO2 and 99-fold in KO4 (*n* = 3; *p* < 0.0001 for all; Figure 3A), whereas Snail mRNA levels were 100-fold reduced in both KO2 and KO4 (*n* = 3; *p* < 0.0001 for all; Figure 3A). Zeb1 mRNA expression was reduced by 40-fold in KO2 and by 46-fold in KO4 (*n* = 3; *p* < 0.01 for all; Figure 3A) in comparison to the Panc-1 vector alone. The Wnt-11 mRNA expression level was significantly reduced in Panc-1 miR-21 KO4 (58-fold; *n* = 3; *p* < 0.05; Figure 3A), but there was not a significant reduction in KO2 (35-fold, *n* = 3; *p* > 0.05; Figure 3A). Similarly, in the MiaPaca-2, E-cadherin mRNA levels were significantly upregulated by 349-fold in KO2 (*n* = 3; *p* < 0.01; Figure 3B) and by 476-fold in KO4 (*n* = 3; *p* < 0.001; Figure 3B). Additionally, vimentin mRNA levels were decreased by 71-fold in KO2 and by 99-fold in KO4 (*n* = 3; *p* < 0.01 and 0.001, respectively; Figure 3B), while Snail mRNA levels presented a significant decrease in KO2 (69-fold) and KO4 (80-fold) (*n* = 3; *p* < 0.05 for all; Figure 3B). Zeb1 mRNA levels were suppressed significantly by 49 and 77-fold in KO2 and KO4, respectively (*n* = 3; *p* < 0.01 and 0.001, respectively; Figure 3B), and Wnt-11 mRNA expression levels reduced in both KO2 and KO4 (57-fold and 49-fold, respectively; *n* = 3; *p* < 0.01 for all; Figure 3B). According to immunofluorescence results, E-cadherin was upregulated; Snail and Wnt-11 were downregulated in miR-21 KO clones when compared to control (*n* = 3; Figure 3C). Overall, our data suggest that miR-21 is involved in EMT through suppressing epithelial characteristics in the cells.

### 2.4. miR-21 Kos Diminish Expressions of CSC Markers in PDAC

**Figure 4:**
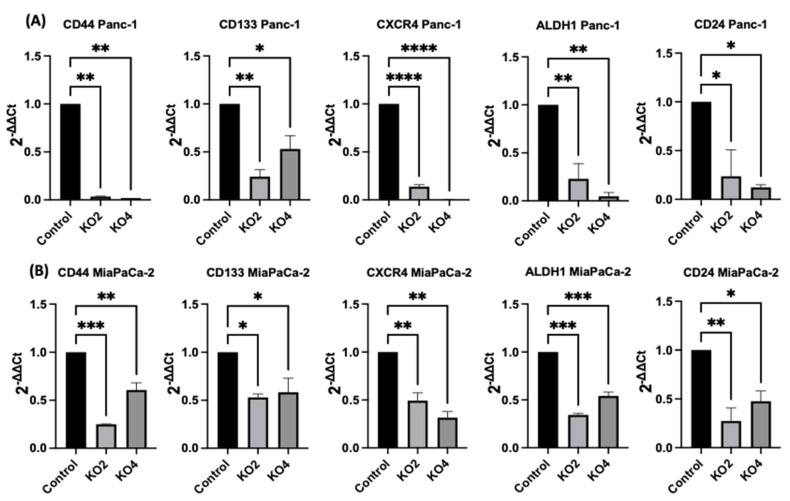
Expression of CSCs markers in PDAC cell lines following miR-21 knockout. (**A**) mRNA expression levels of CD44, CD133, CXCR4, ALDH1, and CD24 in the Panc-1 miR-21 KO cell line, compared with the Panc-1 vector alone. Relative mRNA expression levels of CD44, CD133, CXCR4, ALDH1, and CD24 are significantly reduced in miR-21 KO Panc-1 cells. The column graphic represents the average of three replicates of RNA isolated from Panc-1 control and its Kos. Data normalized according to RPII expression by fold analysis (*n* = 3, *p* < 0.05). (**B**) mRNA expression levels of CD44, CD133, CXCR4, ALDH1, and CD24 in the miR-21 KO MiaPaCa-2 cell line, compared with MiaPaCa-2 vector alone. Relative mRNA expression levels of CD44, CD133, CXCR4, ALDH1, and CD24 are significantly reduced in miR-21 KO MiaPaCa-2 cells. The column graphic represents the average of three replicates of RNA isolated from MiaPaCa-2 control and its Kos. Data normalized according to RNA polymerase II (RPII) expression by fold analysis (*n* = 3, *p* < 0.05). *p*-values are indicated as * *p* ≤ 0.05; ** *p* ≤ 0.01; *** *p* ≤ 0.001; **** *p* ≤ 0.0001; error bars indicate SD.

The expression levels of specific selected CSCs, which are involved in PDAC progression, were assessed in the miR-21 PDAC KO cell lines generated from both Panc-2 and MiaPaCa-2; this included CSC markers CD44, CD24, CD133, CXCR4, and ALDH1. In Panc-1 miR-21 KO clones 2 and 4, the mRNA expression levels of CD44 were decreased by 97% (*n* = 3; *p* < 0.01 for all; Figure 4A), whereas CD133 was downregulated by 76% in KO2 and by 52% in KO4 (*n* = 3; *p* < 0.01 and *p* < 0.05, respectively; Figure 4A). CXCR4 was reduced by 86% in KO2 and by 100% in KO4 (*n* = 3; *p* < 0.0001 for all; Figure 4A), whereas ALDH1 expression levels were reduced by 77% in KO2 and by 95% in KO4 (*n* = 3; *p* < 0.01 for all; Figure 4A). The mRNA expression levels of CD24 were reduced by 77% in KO2 and by 88% in KO4 (*n* = 3; *p* < 0.05 and *p* < 0.05, respectively; Figure 4A), compared with the control Panc-1 vector alone. In the MiaPaCa-2 miR-21 knockout cells, CD44 mRNA expression levels decreased significantly by 75% in KO2 (*n* = 3; *p* < 0.001; Figure 4B) and by 40% in KO4 (*n* = 3; *p* < 0.01; Figure 4B), whereas CD133 mRNA levels were decreased by 50% in KO2 and by 42% in KO4 (*n* = 3; *p* < 0.05 for all; Figure 4B). Similarly, CXCR4 mRNA expression levels were reduced by 51% in KO2 and by 69% in KO4 (*n* = 3; *p* < 0.01 for all; Figure 4B), while ALDH1 mRNA levels decreased by 66% in KO2 and 46% in KO4 (*n* = 3; *p* < 0.001 for all; Figure 4B). The mRNA expression levels of CD24 were significantly reduced by 73% in KO2 (*n* = 3; *p* < 0.01; Figure 4B) and by 53% in KO4 (*n* = 3; *p* < 0.05; Figure 4B). Hence, the findings show that through the knocking out of miR-21 in two PDAC cell lines, the stemness markers CD44, CD133, CD24, CXCR4, and ALHD1 were significantly reduced in both cell lines assessed.

### 2.5. Flow Cytometry Analysis of PCSCs Expression in PDAC miR-21 KO Cells

**Figure 5:**
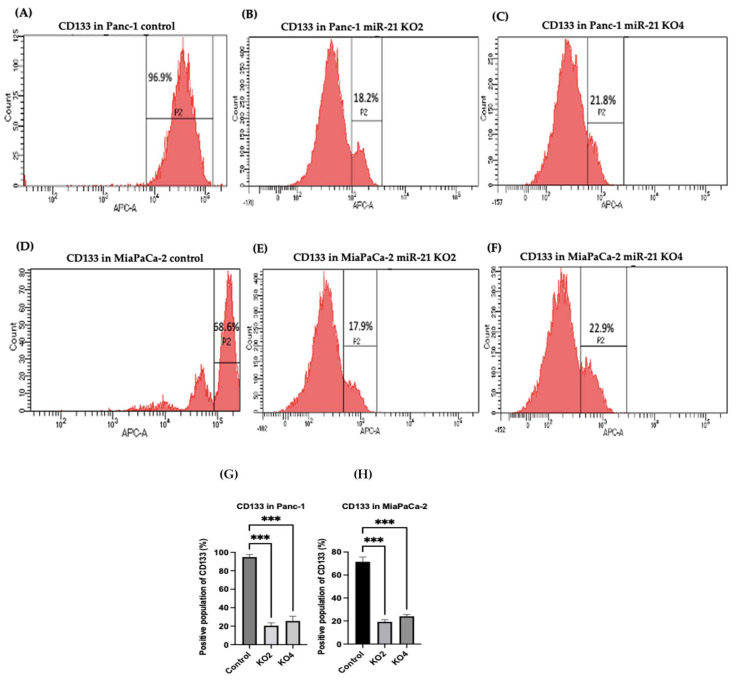
Flow cytometry analysis of CD133 in PDAC control and miR-21 KO cell lines. (**A**–**C**): Expression of CD133 in the Panc-1 miR-21 Kos and control: (**A**) Panc-1 vector alone (control cells); (**B**) miR-21 KO2; (**C**) miR-21 KO4. (**D**–**F**): Expression of CD133 in MiaPaCa-2 miR-21 Kos. (**D**) MiaPaCa-2 vector alone (control cells); € miR-21 KO2; (**F**) miR-21 KO4. (**G**) Percentage of the CD133 positive population in Panc-1 cells and their miR-21 KOs. CD133 expression was significantly increased in the Panc-1 vector alone compared with the miR-21 KOs in the Panc-1 cell line. The column graphic represents the average of three replicates of RNA isolated from Panc-1 control cells and their KOs. Data normalized according to RPII expression by fold analysis (*n* = 3, *p* < 0.05). (**H**) Percentage of the CD133 positive population in MiaPaCa-2 cells and their miR-21 KOs. CD133 expression was significantly increased in MiaPaCa-2 control cells compared with their miR-21 KOs. The column graphic represents the average of three replicates of RNA isolated from MiaPaCa-2 control cells and their KOs. Data normalized according to RPII expression by fold analysis (*n* = 3, *p* < 0.05). Numbers in the gated areas mark the percentages of cells that were positive for this specific marker. *p*-values are indicated as *** *p* ≤ 0.001; error bars indicate SD.

**Figure 6:**
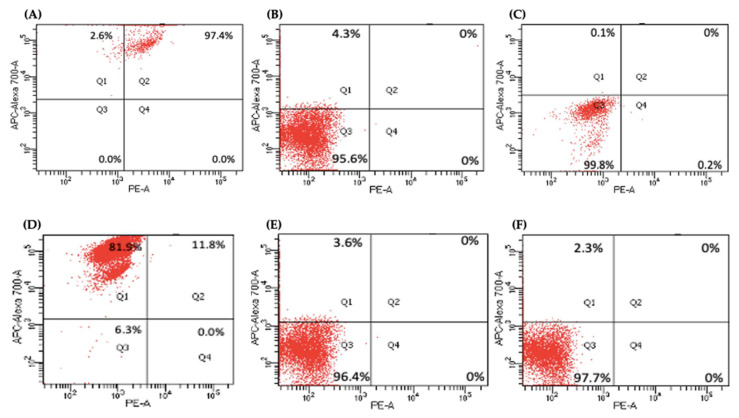
Flow cytometry analysis of CD24 and CD13 in miR-21 KO cell lines. (**A**–**C**) Expression of CD13 and CD24 in the Panc-1 KOs: (**A**) Panc-1 vector alone (control cells); (**B**) miR-21 KO2; (**C**) miR-21 KO4. Expression levels of both CD13 and CD24 were significantly reduced in Panc-1 miR-21 KO2 and KO4, compared with the Panc-1 control cells. (**D**–**F**): Expression of CD13 and CD24 in MiaPaCa-2 KOs: (**D**) MiaPaCa-2 vector alone (control cells); (**E**) miR-21 KO2; (**F**) miR-21 KO4. Expression levels of CD24 were significantly increased in MiaPaCa-2 control cells, while CD13 was significantly reduced not only in MiaPaCa-2 control cells but also in MiaPaCa-2 miR-21 KO2 and miR-21 KO4. APC-Alexa 700 symbolizes the CD24 marker, while PE stands for CD13. Numbers in the gated areas mark the percentages of cells that were positive for this specific marker.

Several key CSCs associated with PDAC, including CD24, CD133, and CD13 were assessed by flow cytometry in the miR-21 KO cells and compared with their expression in control cells. Results showed that CD133 was expressed at higher levels in Panc-1 control cells (96.9%; *n* = 3; Figure 5A), compared with Panc-1 KO2 (18.2%; 74.4-fold reduction; *n* = 3; *p* < 0.001; Figure 5B) and KO4 (21.8%; 69.3-fold reduction; *n* = 3; *p* < 0.001; Figure 5C,G). Similarly, in MiaPaCa-2 miR-21 Kos, the expression levels of CD133 were significantly higher in MiaPaCa-2 control (68.6%; *n* = 3; Figure 5D,H), compared with MiaPaCa-2 KO2 (17.9%; 52.3-fold decrease; *n* = 3; *p* < 0.001; Figure 5E,H) and KO4 (22.9%; 47.5-fold decrease; *n* = 3; *p* < 0.001; Figure 5F,H). Additionally, in the Panc-1 miR-21 Kos, the positive population for CD13 and CD24 presented a 100-fold and 95.7-fold reduction, respectively, in Panc-1 KO2 (0% and 4.3%, respectively; *n* = 3; Figure 6B) and 100-fold reduction in KO4 (0.2% and 0.1%, respectively; *n* = 3; Figure 6C), compared with what was observed in the Panc-1 control, where the positive population for both CD13 and CD24 was 97.4% (*n* = 3; Figure 6A). In the MiaPaCa-2 cell line, control cells were positive for CD24 (81.9%; *n* = 3; Figure 6D) and negative for CD13 (0%; *n* = 3; Figure 6D), whereas the percentage of negative cell population for both CD13 and CD24 PCSCs in the MiaPaCa-2 KO2 was 96.4% (90.1-fold; *n* = 3; Figure 6E) and 97.7% in KO4 (91.4-fold; *n* = 3; Figure 6F; Table 1). These results indicate that the depletion of miR-21 from both PDAC cell lines results in a decrease in the stemness markers CD133, CD24 and CD133.

### 2.6. miR-21 KO Reduce Cellular Invasion in Panc-1 and MiaPaca-2 Cells

**Figure 7:**
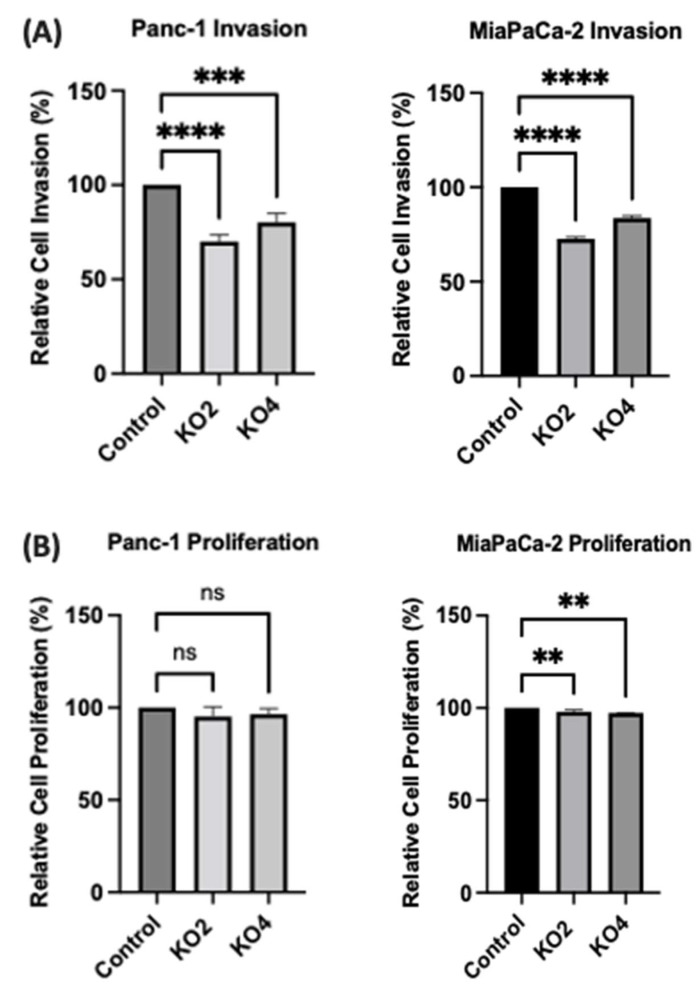

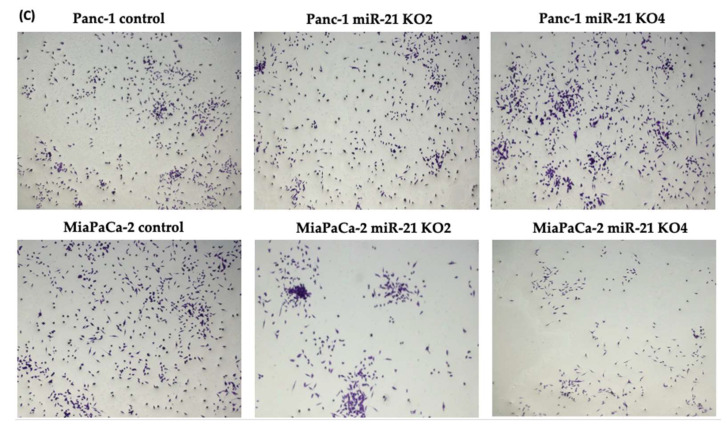
miR-21 reduced cellular invasion of Panc-1 and MiaPaca-2 cells. (**A**) Control and miR-21 KO cells were plated on Matrigel-coated transwell filters and the extent of invasion was determined after 16 h. The results are plotted as Relative Cell Invasion (%), which is the percentage of invaded cells compared to the total number of cells seeded. (**B)** The total cell number/proliferation did not change during the course of the experiment in the Panc-1 miR-21 KOs (*n* = 3; *p* > 0.05 for all), however, a small significant reduction was detected in the MiaPaCa-2 miR-21 KOs (*n* = 3; *p* < 0.01 for all). (**C**) Colony formation assay for Panc-1 and MiaPaCa-2 control and miR-21 KO cells. The colonies were observed with crystal violet staining of cells following 12 days. Images were obtained by using EVOS FL Auto 2 Imaging System (ThermoFisher, UK) with 10x magnification. *p*-values are indicated as ** *p* ≤ 0.01; *** *p* ≤ 0.001; **** *p* ≤ 0.0001; error bars indicate SD.

The invasiveness of both Panc-1 and MiaPaca-2 cells was studied by using Boyden chambers with Matrigel over a 16 h period. KO miR-21 resulted in a significant suppression of invasiveness by 31% and 22% in Panc-1; 27% and 16% in MiaPaca-2 cells (*n* = 3; *p* < 0.0001 and 0.001; *p* < 0.0001, respectively; Figure 7A). There was no significant change in the cell number over the 16 h in Panc-1 cells; however, a significant 3% reduction in MiaPaCa-2 cellular proliferation was detected in miR-21 KOs (*n* = 3; *p* > 0.05; *p* < 0.01, respectively; Figure 7B). The results show that miR-21 KO reduces invasiveness. The Panc-1 miR-21 KO cells did not exhibit a decrease in clonogenicity compared to control cells after 12 days, however, the MiaPaCa-2 miR-21 KO cells presented a reduction in proliferation compared to MiaPaCa-2 control (Figure 7C).

## 3. Discussion

The main results of this study were that: (1) The expression of oncomiRs miR-21, miR-155, miR-221, and tumor suppressor miR-126 is dysregulated in PDAC cell lines compared to normal human ductal epithelial cells; (2) miR-21 moderates mRNA expression levels of key EMT markers, Wnt-11 expression, and cancer stem-like markers; (3) miR-21 KO significantly reduces cellular invasion capability of the two PDAC cell lines studied, indicating a role for miR-21 in cellular invasion capacity of PDAC in vitro.

PDAC remains one of the main fatal malignancies with no specific biomarker for early diagnosis to date [68]. However, in the last few years, several reports have suggested that miRs expression levels can be used as biomarkers to screen for PDAC and its prognosis [69]. High levels of miR-21 expression have been detected in numerous cancers and PDAC, miR-21-5p obtained the highest specificity and sensitivity as an early PDAC diagnostic marker out of seven key candidate miRs identified [70,71]. In the current study, we reported that miR-21 expression was significantly dysregulated in two of the PDAC cell lines, namely Panc-1 and MiaPaCa-2, compared to normal HPDE cells. However, miR-21 expression levels did not significantly change in the PDAC cell line, BxPC3. Overexpression of miR-21 has previously been associated with an elevated proliferation and invasion capability, decreased apoptosis of PDAC cells, chemo/radio-resistance, and uncontrolled renewal of cancerous stem cells [72]. Overexpression of miR-221 has been shown to play a significant role in platelet-derived growth factor (*PDGF*)-mediated EMT phenotype, migration, metastasis, and uncontrolled proliferation of PDAC cells [73]. Importantly, when assessing the expression levels of different miRs in the current study, miR-221 was identified as being upregulated in both Panc-1 and MiaPaca-2 PDAC cells. In addition, miR-221 has been found to lead to the minimization of stem cell repopulating activity in cord blood CD34^+^ cells by targeting *KIT*, while they also act as inhibitors in the proliferation process of erythroleukemia cell lines [74]. 

Increased levels of miR-155 have previously been reported in PDAC patients compared with normal pancreatic tissues and have been shown to suppress the pro-apoptotic gene p53 (*TP53INP1*), which plays a crucial role in p53 function, in inducing growth inhibition and autophagic cell death, in the repression of tumor cell migration, in cell growth arrest and apoptosis [75]. Furthermore, previous reports have also shown that overexpression of miR-155 is linked to the clinical stage (especially PanIN-2 and PanIN-3), lymph node metastasis, and prognosis in PDAC patients [76,77,78,79]. In our current study, elevated expression of miR-155 was found in all three PDAC cell lines assessed, and this aligns with previous findings in PDAC patients highlighted above. The downregulation of miR-126 in PDAC has been reported in previous studies, and this correlates with the findings of our current study, which noted reduced miR-126 expression in the PDAC cell lines assessed [77]. In particular, we found that miR-126-5p was strongly decreased in BxPC3, MiaPaCa-2, and Panc-1 cells by 57%, 81%, and 94%, respectively, indicating a strong correlation between miR-126 and PDAC development. miR-126 is known to inhibit CXCR4, which suppresses cell proliferation, migration, invasion, cell apoptosis, and arrests the cell cycle at the G_0_/G_1_ transition of PDAC cells [80,81]. CXCR4 is a putative mediator between miR-126 and the RhoA/ROCK signaling pathway [80,82], promoting mitogen-activated protein kinase (MAPK) p42/44 phosphorylation and activation of the phosphoinositide-3-kinase (PI3K)/AKT pathway [83,84,85], which are further linked to lymph node metastasis and the unfavorable overall survival of PDAC patients [80].

**Figure 8:**
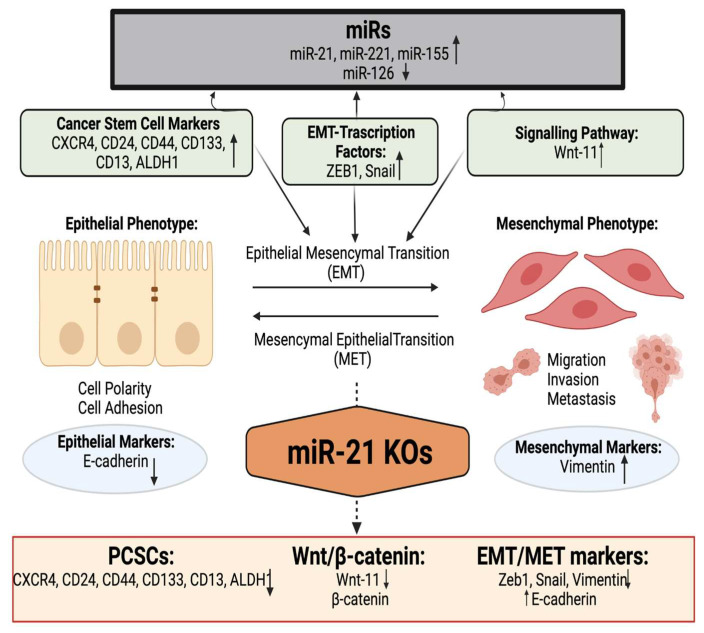
Overview of the involvement of miR-21 in cancer stemness and EMT in PDAC. According to our data, miR-21 affected cancer stemness, EMT, and the Wnt-11 pathway. Created with BioRender.com (accessed on 3 December 2021).

Moreover, in the current study, the EMT-associated markers E-cadherin, Vimentin, Snail, Zeb1, and Wnt-11 were found to be controlled by miR-21 and their mRNA expression levels were significantly affected by miR-21 knockouts in the PDAC cells (Figure 8). Furthermore, immunofluorescence results indicated that *E-cadherin* expression was gained, while *Snail* and *Wnt-11* expressions were reduced in Panc-1 miR-21 KO2 clones when compared to wt/control (Figure 3C). Several studies have disseminated that a number of miRs can promote EMT and cancer stemness in PDAC [86,87,88,89,90,91,92,93,94,95,96], whereas our current study provides some new pilot insights into the role of miR-21 depletion in cancer stemness, EMT, and the Wnt-11 pathway. 

We detected that key CSC markers were dependent on miR-21 status in PDAC cell lines. PCSCs, such as CD24, CD44, CD133, EpCAM, ESA, tyrosine-protein kinase Met (c-met), ALDH1, leucine-rich repeat-containing receptor (lgr5), and serine/threonine-protein kinase (Dclk1), are upregulated during PDAC progression [97,98]. The results of our current study showed that miR-21 regulates not only EMT pathways but also seems to play an essential role in CSCs expression, including CD44, CD24, CD133, CD13, ALDH1, and CXCR4. This is consistent with previous studies that have revealed that the capability of these CSCs for self-renewal can be affected by several miRs, including miR-99a, miR-100, miR-125b, miR-192, and miR-429 [99,100]. Additional research has highlighted interactions between 210 miRs and 258 stem cell-related mRNAs, commonly dysregulated in the PCSCs [101]. Furthermore, the loss of miR-34 has been observed in CSCs, while its restoration can suppress the spheroid-forming ability via the repression of *Notch* and *Bcl-2,* and restoration of *p53* [102]. Importantly, recent studies have shown that PCSCs have contributed to crosstalk with the PDAC parenchymal cells through a symbiotic association, leading to early PDAC infiltration and metastasis [103]. Additionally, other reports showed that cell-surface markers, including CD133, CXCR4, EpCAM, CD24, CD44, ABCG2, and c-Met, have been detected to be upregulated in PDAC cases, and specifically, CD44^+^ CD24^+^ EpCAM^+^ cells were associated with a 100-fold increase in tumorigenic potential compared to CD44− CD24^−^ EpCAM^−^ cells [22,104,105,106,107,108,109]. CD24 contributes to cell adhesion and in the development of organs, such as the brain and kidneys [110,111], while also being closely associated with PDAC [112]. In previous studies, the absence of CD24 has been observed in normal PDAC tissue, while elevated expression levels were observed in the progression from normal ductal epithelium to invasive intraductal papillary mucinous neoplasm (IPMN) [113]. Our data showed that CD24 was highly overexpressed in the MiaPaCa-2 and Panc-1 cell lines and decreased in miR-21 KOs, which suggests that miR-21 can affect stemness. Moreover, CD24^+^ population is associated with higher tumor stage, nodal metastasis, higher-grade tumors, microscopic lymphatic, venous and neural invasion in PDAC [107], and reduction in CD24 expression, as observed here, in response to miR-21 KO in PDAC cells may be of significant importance.

CD133 is a transmembrane protein present in lipid rafts, which has been found to play a crucial role in PDAC tumorigenesis [114,115,116,117]. Recent studies have shown that Wnt/β-catenin signaling is involved in modulating PDAC progression and in promoting self-renewal of CD133^+ ^cancer cells [118,119,120]. Importantly, these findings appear to be well supported by another study, which revealed that CD133 expression levels are considerably decreased in normal pancreatic tissue (only 0.01% of cancer cells), compared to PDAC, where the population of this CSC was 0.5–1% of CD133^+^ cells in less aggressive cell lines to more than 9% of CD133^+^ cells in clones with high migration [121,122,123]. Furthermore, a correlation between EMT and PDAC development has been described through the regulation of the NF-κB signaling pathway, which is activated by CD133 [124,125]. CD133 can therefore induce EMT, while high expression of this marker is correlated to increased proliferation, metastasis of lymph nodes, reduced apoptosis, and tumorigenesis with chemotherapeutic resistance in PDAC cells [126]. Moreover, the presence of CD133 subpopulation in a study where CSCs from 11 primary human PDAC samples and PDAC cell lines was demonstrated, resulted in the reconstitution of PDAC growth and differentiation [104]. Based on this information, the results of the current study, which showed a significant reduction in CD133 in response to miR-21 KO in both PDAC cell lines assessed, indicates the importance of miR-21 in the regulation of this key tumorigenic PDAC protein.

CD44 contributes to cellular adhesion, angiogenesis, and the release of cytokines during PDAC progression [127,128]. Elevated CD44 is linked to EMT-related mesenchymal cancer cell phenotypes [129,130,131] and to increased levels of several mesenchymal markers, as well as high grade of PDAC, including via the activation Akt pathway, which targets E-cadherin expression and thus generates EMT [132]. CD44 overexpression furthermore induces expression levels of transcription factors, including Nanog, Sox2, and Oct4, which further promotes miR-302 and miR-21 upregulation and regulates cell growth/self-renewal elevation in CD44^high^ PDAC cells [133,134,135,136]. A recent study has suggested that the interaction between CD44 and hyaluronan results in the promotion of miR-21 expression, which further leads to the elevated expression of anti-apoptotic protein Bcl-2 [137,138]. In our study, we found that miR-21 moderated the expression levels of several CSCs, including CD44. CD44 indicates reduced stemness in Panc-1 and MiaPaCa-2 cell lines following miR-21 KO. Upregulation of CXCR4 is accepted to be indicative of shorter overall survival and related to an elevated risk of developing lymph node and liver metastasis via the interaction with CXCL12, which can further promote angiogenesis and the formation of new blood and lymphatic vessels [106]. Previously, it has been reported that CXCR4 is involved in PDAC pathogenesis [139,140,141]. This correlates with our data, as we noted that the overexpression of CXCR4 in PDAC cell lines was associated with miR-21 and CXCR4 was significantly reduced in both PDAC cell lines upon miR-21 KO. 

ALDH-1, a CSCs marker, is correlated to tumorigenic cells in PDAC [25,142,143,144]. We found that the high levels of ALDH-1 expressed in control PDAC cells were significantly reduced in response to miR-21 KO in both Panc-1 and MiaPaCa-2 cells. CD13 was another PCSCs marker assessed in the miR-21 KO PDAC cells. PDAC patients with more CD13^high^ neutrophil-like heterogeneous myeloid-derived suppressor cells (nMDSCs) have presented a shorter overall survival than those with fewer CD13^high^ nMDSCs [145,146,147]. Moreover, numbers of CD13^high^ nMDSCs decreased after tumor resection of PDAC cases, whereas CD13^low^ nMDSCs were elevated [146,147]. CD13 MDSCs could be attributed to perineural invasion (PNI) of PDAC, whereas it was also noted that CD13^high^ nMDSCs revealed increased expression levels of Arg1 compared to CD13^low^ nMDSCs, which resulted in more vigorous immunosuppressive activity [146,147]. This underlines the significance of the results of our current study, which indicated that the CD13^+^ population was higher in Panc-1 control cells compared with the miR-21 KOs, while in MiaPaCa-2 cells, CD13 levels were low both in the control and KOs. This is of considerable interest also as Panc-1 is considered the more aggressive PDAC cell line of these two. Therefore, CD13 could be potentially used both for PDAC diagnosis and targeted PDAC treatment, the expression levels of CD13 were upregulated only in the metastatic Panc-1 cell line and not in the MiaPaCa-2 cell line and its two KOs clones.

The results of our current study showed that the miR-21 KOs significantly reduced cell invasion in both Panc-1 and MiaPaca-2 cells, while no significant changes in cell proliferation of Panc-1 cells were observed. miR-21 KOs led a small, however significant, reduction in MiaPaCa-2 cell proliferation over a 16h. These findings are consistent with our previous in vitro studies, which have identified that Wnt-11 is closely associated with cellular invasion of Panc-1 cells [148] and that miR-21 regulates Wnt-11 expression levels not only in PDAC but also in triple-negative MDA-MB-231 breast cancer cells [149]. Similarly, previous reports have indicated that transfection with miR-21 precursors can stimulate invasion, extravasation, and metastasis in cellular models of PDAC [150]. According to the TCGA database, the survival ratio of the low expression cohort (median 22.2 months) was longer than in the high expression cohort (median 19.77 months), based on data from 178 patients. The high expression profile of miR-21 (113 people with high expression of miR-21, compared with 65 people with low expression of miR-21) was significantly correlated with overall survival (Figure S1).

## 4. Materials and Methods

### 4.1. Cell Culture and CRISPR/Cas9 Assay

Panc-1 (ATCC^®^ CRL-1469™), MiaPaCa-2 (ATCC^®^ CRL-x1420™), BxPC-3 (ATCC^®^ CRL-1687™), and non-tumorigenic human pancreatic ductal epithelial cell line (HPDE; H6c7, ATCC^®^ CRL-4023) cell lines were cultured according to ATCC’s recommendations, to 80% confluence in 75 cm^2^ flasks in complete Dulbecco’s Modified Eagle’s Medium (DMEM), with 10% fetal bovine serum (FBS) at 37 °C with 5% CO_2_.

The lentiviral CRISPR/Cas9-mediated miR-21 gene editing vectors encoding four different gRNAs, eGFP (control), and Cas9 protein was kindly provided by Dr. Junming Yue, University of Tennessee Health Science Center, USA, and produced based on the recommendations of previously published studies [149,151,152]. Stable cell lines were generated by transducing the MiaPaca-2 and Panc-1 cells with the lentiviral CRISPR/Cas9 miR-21 gene editing vectors and selection in puromycin (1–10 μg/mL).

### 4.2. RNA Extraction and RT-qPCR 

Total RNA was isolated from Panc-1, MiaPaCa-2, BxPC-3, and HPDE (stored at −80 °C) using RNAzol^®^ RT (Sigma, Hertfordshire, UK). Specifically, cells were isolated by centrifugation at 500× *g* for 5 min, then lysed in 0.5 mL of RNAzol and allowed to stand for 15 min at room temperature. Then lysed cells were centrifuged at 12,000× *g* for 15 min at room temperature; the supernatant was mixed with an equal volume of 100% isopropanol to precipitate RNA, let stand for 10 min, and then centrifuged at 12,000× *g* for 10 min at room temperature. DNA digestion was performed by using a RNase-free DNase set (Qiagen, Manchester, UK), according to the manufacturer′s instructions. Briefly, we added 10 μL DNase I stock solution to 70 μL Buffer RDD, after mixing with pipetted 80 μL DNase I and incubating at room temperature for 15 min. Then, RNA pellets were washed twice with 0.5 mL 75% ethanol (*v*/*v*) per 1 mL of supernatant and centrifuged at 8000× *g* for 3 min at room temperature. The alcohol solution was removed with a micropipette, the RNA pellet was solubilized in RNase-free water, and samples were vortexed at room temperature for 3 min. The RNA concentration was measured using the NanoDrop Spectrophotometer (ThermoFisher Scientific, Hemel Hempstead, UK) at 260 nm and 280 nm absorbance. Moreover, the qScript microRNA cDNA Synthesis Kit (Quantabio, Lutterworth, UK) was utilized to reverse-transcribed RNA cDNA synthesis, according to the manufacturer’s instructions. The resulting cDNA from PDAC cell lines was further used to examine the expression levels of miR-21-5p, miR-221-5p, miR-155-5p, and miR-126-5p, whereas RNU6 was used as a reference gene for the normalization of miRs expression levels. The PerfeCTa SYBR Green SuperMix (Quantabio, Lutterworth, UK) was used with MystiCq miR qPCR primers for the examined miRs purchased from Sigma (Paisley, UK). The following thermocycling conditions were used: denaturation at 95 °C for 2 min, followed by 40 cycles of 95 °C for 5 s, 60 °C for 15 s, and extension at 72 °C for 15 s.

cDNAs to assess the mRNA expression levels of E-cadherin, Vimentin, Snail, Wnt-11, Zeb1, CD44, CD133, CXCR4, and ALDH1 were isolated using qScript™ cDNA Supermix (Quantabio, Lutterworth, UK) with incubations at 25 °C for 5 min, 42 °C for 30 min and 85 °C for 5 min. Precision^®^Plus qPCR Master Mix (Primer Design, Chandler’s Ford, UK) was used for RT-qPCR synthesis for the assessed EMT and CSCs markers with the following thermocycling conditions for 40 cycles: 95 °C for 2 min, 95 °C for 10 s, and 95 °C for 60 s. Relative levels of mRNA expression were calculated as described before [149]. The primers for Snail, Wnt-11, and E-cadherin, were designed and purchased from Sigma (Paisley, UK), Vimentin, and Zeb1 from Integrated DNA Technologies (IDT) (Leuven, Belgium), while CD133, CD24, CD44, CXCR4, and ALDH1, from ThermoFisher Scientific (UK). Primer sequences are presented in Table 2. Relative levels of mRNA expression were calculated using the comparative CT/2^−ΔΔCt^ method [153] with RPII as the reference gene for the in-cell-line-based studies. In addition, the standard deviation was calculated as well as a *t*-test using GraphPad Prism 7.00 (La Jolla, CA, USA) software.

### 4.3. Immunostaining

Panc-1 wt (Control) and miR-21 KO cells were seeded into 6-well plates and allowed to settle overnight; the cells were washed with 1× PBS and fixed with 4% formaldehyde for 20 min at room temperature. Cells were washed with 1× PBS twice, following blocking with 5% *w*/*v* bovine serum albumin (Invitrogen, Loughborough, UK) for 30 min at room temperature. The wells were washed with PBS twice and primary antibodies E-cadherin, Snail (20C8) (Invitrogen, Waltham, MA, USA), and Wnt-11 (GeneTex, CA, USA) were added and incubated for one hour. After washing the cells, either goat anti-rabbit IgG Alexa Fluor or anti-mouse IgG (ThermoFisher, Oxford, UK) was added and incubated for an hour. Following another washing step with 1× PBS, Ribonuclease A 100 mg/mL (Sigma–Poole, Dorset, UK) was added and incubated, gently rocking for 20 min. For counterstaining, 5 µL/mL of 1 nM To-Pro-3 (ThermoFisher Scientific, Oxford, UK) was dispensed into each well and set gently rocking, and then washed twice with PBS for 5 min gently rocking. The results generated were taken from the three biological and technical repetitions. Three to four milliliters of 1× PBS was added to each well, Leica TCS SP2 (Leica Microsystems; Milton Keynes, UK) confocal microscope was used to analyze the cells.

### 4.4. Flow Cytometry Analysis

Cells (seeding density 2 × 10^5^ in each well/6-well plate) were pelleted by centrifugation for 5 min at 500× *g*. The supernatant was removed, and cells were resuspended in 10 μL of CD133 (APC Mouse Anti-Human CD133), CD13 (CD13 PE), and CD24 (Alexa Fluor^®^ 700 Rat Anti-Mouse CD24) antibodies (BD, Berkshire, UK) and incubated for 30 min on ice in the dark. Then cells were washed twice with 1× PBS for 5 min at 500× *g* and resuspended in 200 μL of 1X PBS. Results were analyzed by a flow cytometer using C6 software (BD LSRFortessa X-20; BD, Berkshire, UK).

### 4.5. Matrigel Invasion and Proliferation Assays

Matrigel cell invasion assay was performed as described previously [68]. Briefly, 5 × 10^5^ cells were plated on Matrigel-coated transwell filters (Matrigel™ Invasion Chamber, Corning; BD Biosciences, Wokingham, Berkshire, UK) in a chemotactic gradient of 1:10% FBS. In parallel, the same number of cells was plated and incubated for 16 h to determine the cell proliferation by a MTT (3-(4,5-dimethylthiazol-2-yl)-2,5-diphenyl tetrazolium bromide) assay. After 16 h incubation, the total number of invaded cells was determined by a MTT assay (Abcam, Cambridge, UK). Absorbance was measured using a CLARIOstar plate reader (BMG Labtech, Aylesbury, UK) at 540–590 nm and normalized according to the control. The experiments were performed three times from different biological samples with 3 technical repeats.

### 4.6. Colony Formation

Briefly, both MiaPaCa-2 and Panc-1 cells transduced with lentiviral miR-21 gRNA and control vectors were seeded at 1 × 10^4^ density in 6-well plates and incubated for 12 days. After the media was removed and cells were washed with 1× PBS solution, they were fixed with methanol: acetic acid (3:1) for 20 min at room temperature. After removing the fixing agent, cells were stained with 0.5% *w*/*v* crystal violet in methanol for 15 min, cells were washed with distilled water, and cells were imaged using an EVOS FL Auto 2 Imaging System (ThermoFisher, Hemel Hempstead, UK).

### 4.7. Statistical Analysis

The expression levels of different miRs, proteins, and CSCs in PDAC cell lines were examined using ANOVA and Bonferroni multiple comparisons tests followed by Tukey’s post-hoc analysis. Specifically, free commercially available software packages GraphPad Prism v8.4 (La Jolla, CA, USA) was utilized for the statistical analysis. Statistical significance was conducted as Tukey at *p* ≤ 0.05, while all the results were presented as mean ± SD.

## 5. Conclusions

In conclusion, our data indicate that miR-21 regulates key CSC markers and affects EMT markers in PDAC. The EMT and Wnt-11 pathway was found to be modulated by miR-21 knockout, highlighting the importance of miR-21 as a potential target of cancer stemness. While further and in-depth studies will be needed to identify all related mechanisms for the role of miR-21 in the poor prognosis and metastasis of PDAC; the data presented in this study provide novel insights into roles for miR-21. Furthermore, our data support previous findings that show the importance of miR-21 and its potential as a therapeutic target for PDAC.

## Figures and Tables

**Table 1:** Percentage of expression of CD24 and CD13 in PDAC Cell Lines.

Cancer Stem Cell Markers	Control	Panc-1 KO2	KO4	Control	MiaPaCa-2 KO2	KO4
CD24^+^ CD13^+^	97.4%	0%	0%	11.8%	0%	0%
CD24^−^ CD13^−^	0%	95.6%	99.8%	6.3%	96.4%	97.7%
CD24^+^ CD13^−^	2.6%	4.3%	0.1%	81.9%	3.6%	2.3%
CD24^−^ CD13^+^	0%	0%	0.2%	0%	0%	0%

**Table 2:** Sequences of primers used in the study.

Primer	Forward Sequence (5′–3′)	Reverse Sequence (5′–3′)
E-cadherin	AAGAAGCTGGCTGACATGTACGGA	CCACCAGCAACGTGATTTCTGCAT
Wnt-11	GTGAAGGACTCGGAACTCGT	CTTCTGTTCCTGGTGGCTTC
Snail	TTTCTGGTTCTGTGTCCTCTGCCT	TGAGTCTGTCAGCCTTTGTCCTGT
Vimentin	TACAGGAAGCTGCTGGAAGG	ACCAGAGGGAGTGAATCCAG
Zeb1	GGGAGGAGCAGTGAAAGAGA	TTTCTTGCCCTTCCTTTCTG
U6	GCTTCGGCAGCACATATACTAAAAT	CGCTTCACGAATTTGCGTGTCAT
RPII	GCACCACGTCCAATGACAT	GTGCGGCTGCTTCCATAA
CD133	AAGCATTGGCATCTTCTATGG	AAGCACAGAGGGTCATTGAGA
CD24	GAAAACTGAATCTCCATTCCACAA	TGAAGAACATGTGAGAGGTTTGAC
CD44	CCAGAAGGAACAGTGGTTTGGC	ACTGTCCTCTGGGCTTGGTGTT
ALDH1	ATCAAAGAAGCTGCCGGGAA	TCTTAGCCCGCTCAACACTC
CXCR4	GCCAACCATGATGTGCTGAAAC	GCCAACGTCAGTGAGGCAGA

## Data Availability

Data are contained within the article.

## Supplementary Materials

The following are available online at https://www.mdpi.com/article/10.3390/ijms23031275/s1.

## Abbreviations

ALDH-1	Aldehyde dehydrogenase-1
CA 19-9	Carbohydrate antigen 19-9
CSCs	Cancer stem cells
CXCR4 00	CX-C chemokine receptor type 4
DMEM	Dulbecco’s Modified Eagle’s Medium
EMT	Epithelial–mesenchymal transition
FBS	Fetal bovine serum
FoxM1	Forkhead box protein M1
HPDE	Human pancreatic ductal epithelial cell line
IDT	Integrated DNA Technologies
IPMN	Intraductal papillary mucinous neoplasm
KOs	Knockouts
MAPK	Mitogen-activated protein kinase
MDSCs	Myeloid-derived suppressor cells
miRs	microRNAs
mTOR	Mammalian target of rapamycin
nMDSCs	Neutrophil-like heterogeneous myeloid-derived suppressor cells
NF-κB	Nuclear factor kappa-light-chain-enhancer of activated B cells
oncomiR	Oncogenic miR
PanIN	Pancreatic intraepithelial neoplasia
PCa	Pancreatic cancer
PCSCs	Pancreatic cancer stem cells
PDAC	Pancreatic ductal adenocarcinoma
PDCD4	Programmed cell death 4
PDGF	Platelet-derived growth factor
PI3K	Phosphoinositide-3-kinase
PNI	Perineural invasion
RPII	RNA polymerase 2
SD	Standard Deviation
SHH	Sonic Hedgehog
TNM	Tumor-Node-Metastasis
UTRs	Untranslated regions
VEGF	Vascular endothelial growth factor
ZEB1	Zinc Finger E-box binding homeobox 1
