# Supplementary figures and images for: microRNA-21 Regulates Stemness in Pancreatic Ductal Adenocarcinoma Cells
Source: Int J Mol Sci. 2022 Jan 24;23(3):1275. doi: 10.3390/ijms23031275 (PMC8835847; doi:10.3390/ijms23031275)

## Slide 1
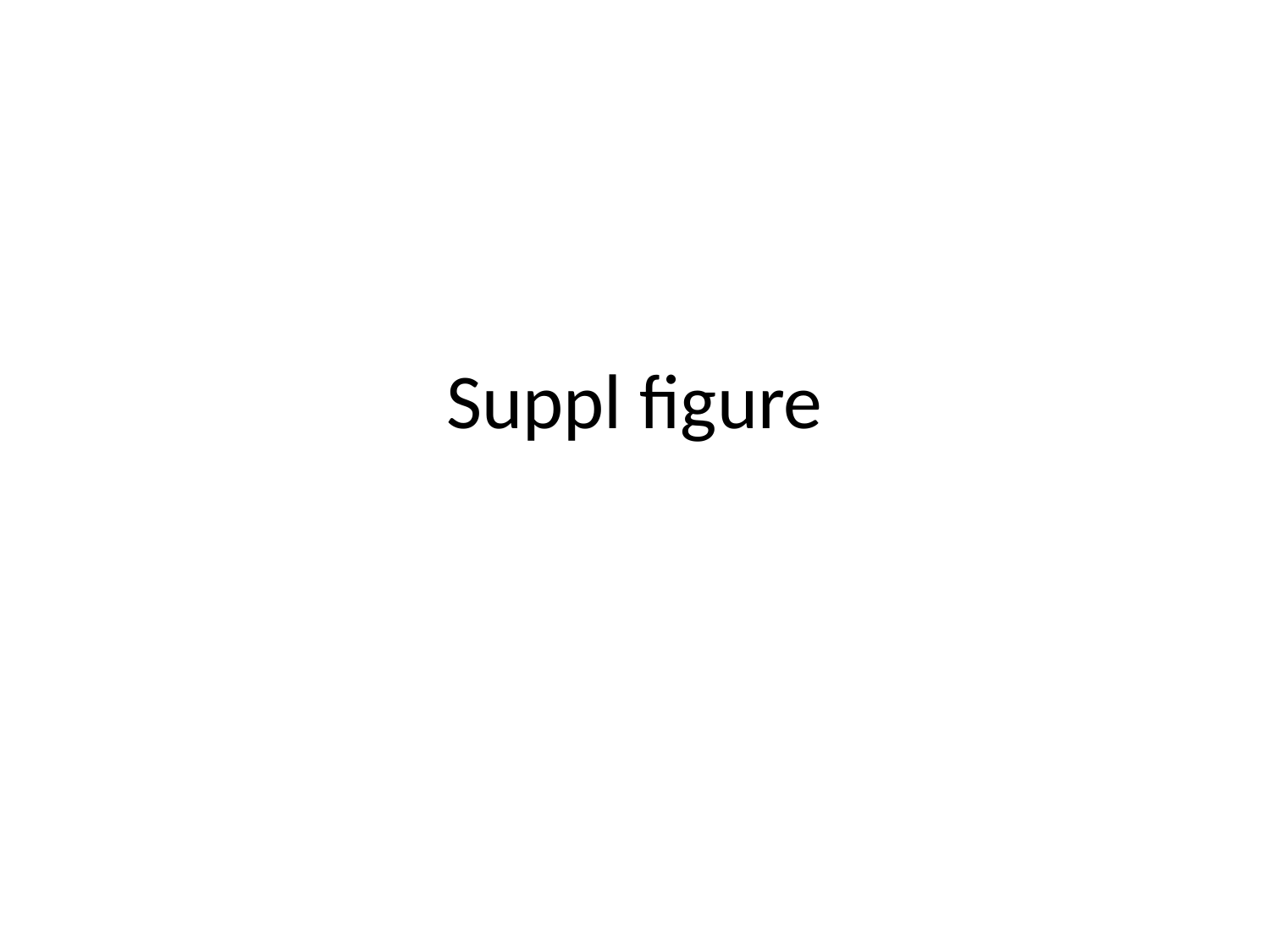

# Suppl figure

## Slide 2
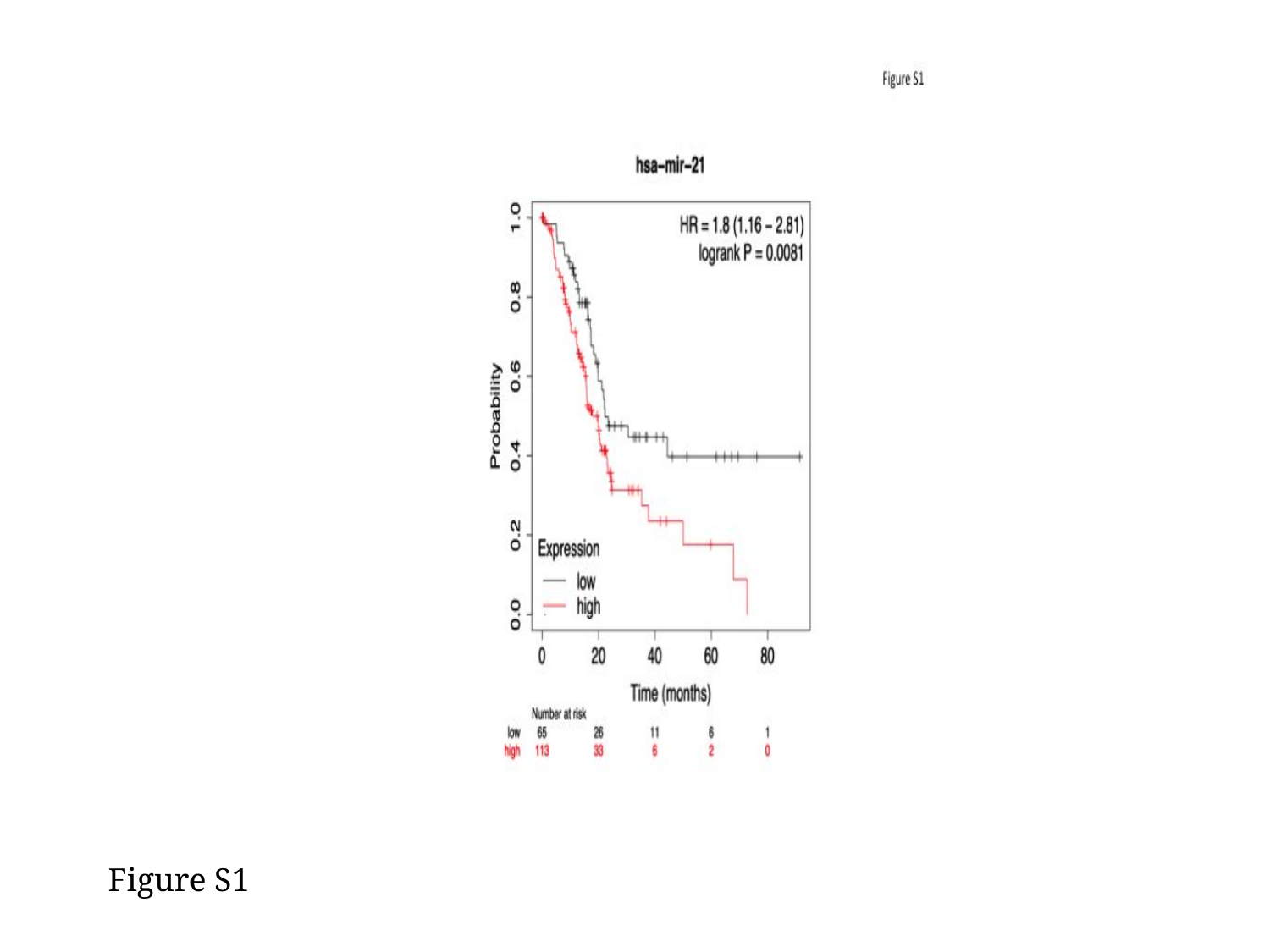

Figure S1

Supplement: Supplementary file 1 [file ijms-23-01275-s001.zip › ijms-1537771-supplementary.pptx]
